# Discrimination of Multiple Coronal Stop Contrasts in Wubuy (Australia): A Natural Referent Consonant Account

**DOI:** 10.1371/journal.pone.0142054

**Published:** 2015-12-03

**Authors:** Rikke L. Bundgaard-Nielsen, Brett J. Baker, Christian H. Kroos, Mark Harvey, Catherine T. Best

**Affiliations:** 1 MARCS Institute and School of Humanities & Communication Arts, Western Sydney University, Sydney, Australia; 2 La Trobe University, Melbourne, Australia; 3 University of Melbourne, Melbourne, Australia; 4 Curtin University, Perth, Australia; 5 University of Newcastle, Newcastle, Australia; 6 Haskins Laboratories, New Haven, Connecticut, United States of America; Leiden University, NETHERLANDS

## Abstract

Native speech perception is generally assumed to be highly efficient and accurate. Very little research has, however, directly examined the limitations of native perception, especially for contrasts that are only minimally differentiated acoustically and articulatorily. Here, we demonstrate that native speech perception may indeed be more difficult than is often assumed, where phonemes are highly similar, and we address the nature and extremes of consonant perception. We present two studies of native and non-native (English) perception of the acoustically and articulatorily similar four-way coronal stop contrast /t ʈ $\underset{̪}{\text{t}}$ ȶ/ (apico-alveolar, apico-retroflex, lamino-dental, lamino-alveopalatal) of Wubuy, an indigenous language of Australia. The results show that all listeners find contrasts involving /ȶ/ easy to discriminate, but that, for both groups, contrasts involving /t ʈ $\underset{̪}{\text{t}}$/ are much harder. Where the two groups differ, the results largely reflect native language (Wubuy vs English) attunement as predicted by the Perceptual Assimilation Model [1, 2, 3]. We also observe striking perceptual asymmetries in the native listeners' perception of contrasts involving the latter three stops, likely due to the differences in input frequency. Such asymmetries have not previously been observed in adults, and we propose a novel Natural Referent Consonant Hypothesis to account for the results.

## Introduction

In phonological and psycholinguistic theory, the speech sounds of a language constitute distinct categories in a speaker's mental grammar: the phonemes of the language. One reasonable assumption might be that all such contrastive phonemes should be equally distinct within that phonological system, and we would therefore expect native listeners to be equally able to discriminate between any pair of phonemes in their own language. However, it has long been acknowledged that this is not always the case (e.g. [4, 5, 6, 7, 8]). Evidence from speech perception research as well as research on language typology, language acquisition, and language change reviewed below supports the viewpoint that discrimination performance levels can and do vary among native speech contrasts. The extent and nature of native perceptual difficulty, however, is unclear, and the issue has been given little attention in theories of speech perception or phonology. If indeed the phonemes of a language do *not* constitute an equally perceptible set, this would have fundamental ramifications for the representation of language in the brain, and for language evolution, change, and acquisition.

In the following, we present a two-part study of native and non-native listeners’ (Australian English) perception of the coronal stop series of Wubuy (an indigenous language of Australia), which includes contrasts that are likely to vary in discriminability for non-native listeners and even for native listeners. Wubuy is ideally suited to address this issue because it employs a very rare four-way coronal stop contrast /t ʈ $\underset{̪}{\text{t}}$ ȶ/—apico-alveolar, apico-retroflex, lamino-dental and lamino-alveopalatal—which we argue is a rather extreme case of phonological distinctiveness coupled with acoustic and articulatory similarity. To our knowledge, this is the first systematic examination of native and non-native discrimination of a four-way coronal stop contrast in any language to date. The only previous study [9] of the perception of a similar contrast series in Western Arrernte (another indigenous Australian language) found that native speakers were inconsistent in categorising coronal stops /t ʈ $\underset{̪}{\text{t}}\;\underline{\text{t}}$/ (where the symbol '$\underline{\text{t}}$' indicates a lamino-alveopalatal stop, our 'ȶ'), typically confusing apico-alveolar /t/ with apico-retroflex /ʈ/. However, it is not possible to attribute the confusion specifically to the phonetic similarity of the apical contrast because Arrernte shows apical neutralisation in initial position—the typical pattern in languages with this contrast [10]—and thus does not provide the full set of coronal stop contrasts needed to address the issue of interest here. By contrast in Wubuy, the four-way coronal series is contrastive both word-medially and word-initially, though Heath [11] notes that the apical contrast (i.e., /t ʈ/) is difficult to distinguish in the absence of a preceding vowel.

The findings we report here provide direct evidence that native language acquisition does not always result in perfect native speech perception when the contrasting phonemes are acoustically and articulatorily highly similar. We extend the Perceptual Assimilation Model of non-native and second-language (L2) speeech perception (PAM; [1, 2], and PAM-L2 [3]), to situations of 'less-than-perfect' *native* perception, and provide testable hypotheses for such scenarios.

Furthermore, with regard to the issue of variations in discrimination of these contrasts by native listeners, our findings are compatible with the claim in [9] that native [Arrernte] listeners do not consistently identify the difference between retroflex and alveolar stops, but extend beyond this observation in several important ways. As we report, native [Wubuy] listeners have varying difficulties discriminating among all three of the non-palatal coronal stops /t ʈ $\underset{̪}{\text{t}}$/. Crucially, we also find that they display notable perceptual asymmetries among these consonant contrasts: Greater ease of discrimination between two members of a contrast depending on presentation order, suggesting innate settings or perceptual biases as detailed below. This pattern is reminiscent of asymmetries that have been reported for vowel perception in infants and L2 learners [12, 13, 14, 15], for which those authors propose the existence of a set of ‘Natural Referent Vowels’. Accordingly, we put forth a proposal that there exist Natural Referent Consonants, the analogue of Natural Referent Vowels, which can help to account for these observed asymmetries in both native and non-native discrimination of these consonant distinctions.

### 1.1 L1 perception is not always perfect

If phonological distinctiveness were the only thing that mattered to a native perceiver, we would expect speakers of a language to be equally good at discriminating all phonemes of their language, irrespective of what measurable acoustic or articulatory similarities and differences might exist between them. The centrality of phonological distinctiveness to native speech perception is exemplified by the phenomenon of categorical perception, which is characterised by great ease in discriminating between consonants that are contrastive, such as (for English speakers) /t/ and /d/, and of great difficulty in discriminating between acoustically different tokens from the same phonological category, e.g. initial /d/ realized with -30 ms voicing lead (prevoicing) versus initial /t/ realised with 0 ms voicing lead, similar to French, which are both perceived as /d/ by English listeners. If phonological distinctiveness were the only relevant parameter, English phonemes such as /p/ and /n/, which differ in terms of place and manner of articulation and voicing characteristics, should be just as easy or just as difficult for native listeners to discriminate as /p/ and /b/, which differ only in terms of voicing. The same should be true for vowels as well (although they are not perceived as categorically as consonants): Australian English vowels /ɪ/ and /i/, which differ in duration and spectral quality, should be as easy to discriminate for a native speaker as /u/ and /e/.

However, research shows that this is not the case. In particular for vowels, but even in the case of consonants, it has been shown that performance on native language speech perception tests is not always perfect: it can be very difficult to correctly identify native phonemes that are acoustically and articulatorily highly similar. In the absence of lexical information, some English fricatives (e.g., /f/ and /θ/) are notoriously difficult to distinguish, even for native listeners ([4, 5, 16], among others). [17] showed that native speakers of Canadian English often confuse native /ɛ/ for /æ/ (15%), and sometimes /i/ for /ɪ/ (5%), and /ʊ/ for /ʌ/, /u/ and /ɔ/ (between 5–2% respectively), but never confuse /ɪ/, /æ/, /ɜ/ and /a/ for any other vowel. In some cases, native phoneme discrimination may even be poorer than non-native discrimination. [18], for example, compared the discrimination of English /w j/ by native speakers of English and Danish and found that the Danish participants, for whom this is not a native contrast, much more accurately discriminated the contrast than native English speakers. They ascribed the superior non-native performance to the fact that there are many more approximant phones (phonemes and allophones, and several vowel rounding settings) in Danish than in English, providing the Danish listeners with greater opportunities to fine-tune their perceptual system to approximants.

Thus far, native speaker perceptual difficulties in consonants have only been demonstrated for continuant (e.g., fricatives) and sonorant (e.g., approximants) segments, and not for stops. The reasons for this are presumably to do with the differences between categorical and gradient (continuous) perception.

We also find suggestive (indirect) evidence that some phones might be more difficult to perceive in that some phonemes are much rarer than others cross-linguistically. One account for these inequalities is that those which occur more frequently are more differentiated from others acoustically and perceptually, which results in more linguistic redundancy and fewer misperceptions. Conversely, the rarity of certain other contrasts, such as that between dental and alveolar fricatives, may be due to their low perceptual salience and/or discriminability from adjacent consonants [19, 20].

Finally, difficulties in perception of phonological contrasts have been identified as a leading cause of sound change in languages by a number of researchers, in particular [6, 7, 8] as well as others [21, 22, 23]. Notably, in such cases, it is the native speakers of a language who are central to the scenario of perceptual difficulty leading to phonological re-analysis.

These multiple strands of independent research indicate that some native phonemic contrasts are more difficult to categorise and discriminate than others. Such difficulties have not yet been, but need to be, adequately addressed by the leading theories of speech perception. In the following, we show how PAM/PAM-L2 has the necessary conceptual framework to account for such patterns and provide testable hypotheses, if applied in conjunction with our novel proposal of innate perceptual biases in consonant perception: the Natural Referent Consonant hypothesis.

### 1.2 Extending PAM/PAM-L2 to difficult native phonological contrasts

Despite the above observations that some native phonemic contrasts might be more difficult than others to identify and discriminate, current theories of first (L1) and second (L2) language speech perception such as the Perceptual Assimilation Model (PAM; [1, 2]), and its extension PAM-L2 [3], the Speech Learning Model (SLM: [24]), and the Native Language Magnet model (see for instance [25]), have not yet systematically considered the theoretical implications of evidence that some contrasts might be inherently difficult to perceive, even for native speakers. This is reflected in the fact that within the cross-language literature speech perception is rarely tested using native stimuli, *except* to provide a control baseline for comparison with non-native listeners' performance (and here, the expectation, and perhaps requirement, is often that native perception will be near-perfect).

The Perceptual Assimilation Model and PAM-L2, however, are able to account for such a pattern of perception relatively straightforwardly. PAM/PAM-L2 assumes that native consonant perception is categorical, yet at the same time recognises a relationship between categoricity and gradiency in perception. For example, the distinction between the ‘Category Goodness’ (CG) assimilation type versus the ‘Single Category’ (SC) assimilation type (see definitions below) is, in its essence, about the perceptibility of within-category gradient variation.

The tenets of PAM/PAM-L2 allow us to examine the native phonological system of an individual by presenting him or her with phones from a different language that do not align with the phoneme boundaries of the native language of the listener. According to PAM/PAM-L2, non-native phoneme contrasts will be perceived in one of the following ways: a Two-Category (TC) assimilation where the two non-native phones are perceived as instances of two separate native phonological categories and discrimination is excellent; a Category Goodness (CG) assimilation where the two non-native phones are perceived as varying instances of the same native phoneme in which one is more native-like than the other and discrimination is moderate to good, depending on the perceptual distance between the two; Single Category (SC) where the two non-native phones are perceived as equally good (or poor) instances of a single native phoneme, and discrimination is expected to be poor; an Uncategorised-Categorised (UC) assimilation where one non-native phone is perceived as an instance of a native phoneme, but the other phone is not assimilated into any single native phoneme category or perhaps weakly assimilated to two or more categories, and discrimination is expected to be moderate to excellent, depending on the perceptual distance between the two; and finally an Uncategorised-Uncategorised (UU) assimilation where neither non-native phone is assimilated into a single native phoneme category, and discrimination success depends on the perceptual distance and extent of multiple-category overlaps between the two.

No study has hitherto applied PAM/PAM-L2 and the related experimental paradigm to adult *native* speech perception in scenarios where there is a high degree of acoustic and articulatory, and possibly perceptual, similarity between two contrasting L1 phonemes, resulting in less-than-perfect discrimination, even by native listeners. (A number of studies have, however, investigated infant perception of acoustically/articulatorily similar native phones; see for instance [26], and [27, 28, 29, 30]). Indeed, we propose that two distinct consonant categories, if very close together in articulatory and/or acoustic space, may overlap phonetically to such an extent that even native listeners may become unsure of whether they are hearing two distinct phonemes (a TC discrimination of highly similar phones). In the case of the Wubuy alveolar versus retroflex contrast for instance, we argue that the phonetic overlap is so great that native listeners can behave as if this were an instance of a good and a 'less good' instance of a single category (CG). Such a pattern has also been shown for fricatives in English (see references in Section 1.1). Especially if there is no lexical information to assist them, as is often the case in studies of segmental categorisation and discrimination, it is likely that discrimination accuracy will be commensurate with the discrimination accuracy expected for a non-native CG contrast, even for native listeners. As our review of Wubuy below indicates, such a scenario may indeed exist in a number of Australian languages, which have up to four coronal places of articulation for stops.

In cases of extreme perceptual difficulty, such as we are proposing, listeners may rely on universal biases in speech perception, which may be reflected in asymmetries in discrimination performance (i.e., greater ease in discriminating two phones in one presentation order than in the opposite order). Until now, evidence for perceptual asymmetries in discrimination studies, as reflections of universal biases, has been largely confined to studies of vowel perception in L1 infants and L2 adult listeners. A few studies have also found perceptual asymmetries in adult L2 perception of stop-fricative consonants /b v/ [31]. It is unclear, however, to what extent contrasts that differ in *manner* of articulation can inform us with respect to contrasts that differ in *place* of articulation. Finally, there are priming and Event Related Potential (ERP) studies that suggest that coronals may suffer an asymmetry with respect to stops with other places of articulation (labial in particular) [32; 33], though other studies using ERP [34] and eye-tracking [35] suggest that these effects do not provide evidence for a universal bias in the perception of coronals vis-a-vis phones with other places of articulation, but may instead be due to differences in the distribution of these places of articulation within the lexicon and thus to listeners' expectations about the likelihood of a particular place of articulation in a particular position within the stimulus. To our knowledge, perceptual asymmetries in discrimination have not been found, until the current study, in L1 adults, nor in the perception of consonants when manner of articulation is held constant.

Polka and colleagues ([12, 13, 14, 15]; also [36]) observed that infant L1, as well as adult L2, vowel discrimination is much better when the participants are first presented with a more central vowel and then tested for detection of change with a more peripheral vowel. More peripheral vowels appear to act as perceptual 'anchors' for detecting changes from one vowel to another. According to [13], the observed infant L1 asymmetries:

‘… could not be explained either by considering the status of the vowel in the infant's native language (i.e. whether or not the anchor vowel is in the infant's native inventory) nor by referring to universally favored vowels (i.e. predictions based on markedness). These effects were also not consistent with an acoustic bias related to a simple increase or decrease in either F1 or F2 and could not be due to differences in amplitude, duration or pitch […]. However, all of these directional asymmetries could be predicted by considering the relative position of each vowel in the vowel space defined by dimensions of vowel height […] and front/back[…]. Within this space, [..], a vowel change from a more central to a more peripheral vowel is consistently easier to discriminate compared to the same change presented in the reverse direction […]’ (p. 467).

In addition to being crucial to the understanding of potential biases in perception in general, we also believe that a focus on asymmetrical discrimination performance is crucial to understanding the effect that differences in the relative distributional frequency of individual phonemes in the input may have on discrimination of native contrasts involving those phonemes. Indeed, we propose that innate perceptual biases will only be overridden by language specific perception when there is sufficient input, i.e. relatively high frequency of occurrence, to 'reset' the perceptual system. We expand on these issues in the following section, introducing a 'Natural Referent Consonant' Hypothesis first, and then discussing the potential effects on discrimination of differences in phone frequency in the input.

### 1.3 A Natural Referent Consonant Hypothesis

Here, we propose a Natural Referent Consonant (NRC) hypothesis for the study of perceptual asymmetries in consonant perception. We propose that such a framework will add to the general understanding of native and non-native consonant perception spearheaded by models such as PAM/PAM-L2. The NRC hypothesis is compatible with PAM/PAM-L2, and likely also with other speech perception theories.

While the articulatory and acoustic space of consonants may not lend itself to the same notion of peripheries in an acoustic-phonetic space as is crucial to the Natural Referent Vowel hypothesis, the three major articulators (lips, tongue front, tongue back) may provide equivalent perceptual and articulatory ‘anchor points’, corresponding to the three major oral places of articulation: labial, coronal, and dorsal (c.f., [37]). From an acoustic and perceptual point of view, such articulatory anchor points are perhaps reflected in phonemes with excellent differentiation. Indeed, it might be possible to establish quantitatively that in acoustic and perceptual terms, labial, alveolar and velar are maximally differentiated from each other, although we do not pursue this here. Perhaps not surprisingly, the vast majority of the world’s languages use all—*and only—*these three places of articulation in consonants. Only a small percentage has multiple sub-places of articulation (manner held constant) within one of these major places. (We infer this from [19]: ‘In a very high proportion of the world's languages, segments with the same manner must be drawn from different active articulator classes’ (p. 43). However, we note that a contrast between velar and uvular voiceless stops is not uncommon, found in many Afro-Asiatic, Caucasian, North American and Turkic languages [19].) Our target language Wubuy is one of these rare languages with multiple coronal sub-places within the same manner classes.

The NRC hypothesis gives rise to a number of predictions on the basis of the general assumptions that:

1) the most canonical articulation, by which we mean the one occurring most frequently for a given major place across the world's languages, will be the perceptual anchor, and that
2) arguably, the canonical articulation for the coronal place is apical orientation of the tongue tip, with contact made in the alveolar region.

Indeed, the case can be made that this is an 'easy-to-achieve' coronal articulation, which provides excellent acoustic distinctiveness from the surrounding major places of articulation. [38] provides explicit discussion of ‘neutral’ vs ‘displaced’ articulations and argues that dental is the neutral passive articulation for apicals, and alveolar for laminals. Considerations of cross-linguistic frequency suggest a different interpretation, however. According to [19] 'In general, if a language has only a dental or an alveolar stop, then that stop will be laminal if it is dental and apical if it is alveolar' (p. 26; they go on to urge caution with the terms 'dental' and 'alveolar'). In addition, however, they note (op. cit.) that 'if a language has both an apical and laminal stop consonant, then the laminal consonant is likely to be more affricated'. It is unclear whether lamino-postalveolar stops also provide a canonical articulation, partly because obstruents with this articulation tend to be affricated in contrast with apical stops in the same inventory. However, according to the phonological database compiled by [39], languages with a single coronal place far outnumber those with two or more (82% for 1 vs 18% for 2 vs .04 for 3 or more). Together with the point that [19] make (above), we take this to be evidence that there are unlikely to be two canonical coronal articulations.

These biases may (indeed, must) be over-ridden by the phonological system of the native language that an infant learns when this system differs from the biases. However, we propose that they may persist in situations where the input frequency for some of the respective consonant phonemes is too low to provide robustly distinct categories for acoustically and articulatorily highly similar phones. In addition to these relatively straightforward analogues of the Natural Referent Vowel hypothesis, therefore, we suggest that the distributional frequencies of consonants overall, or in particular positions in words (syllabic, morphological), will be relevant to native listeners' discrimination. In particular we propose that:

3) Consonant contrasts in which one member occurs infrequently in the speech input may continue to display the innate bias to the Natural Referent Consonant if the canonical articulation is also the more frequent member of the contrast. By this we suggest that only frequently occurring non-canonical consonants are likely to over-ride a natural initial perceptual asymmetry, while under-represented consonants are more likely to continue to display asymmetrical perception past infancy and completion of L1 acquisition and into adulthood. Below, we propose that this is the case specifically with retroflex stops in Wubuy.
4) Speakers of languages that make use of only the three major oral places of articulation (labial, coronal, dorsal) and that use no distinctions *within* any of the major places for a given manner class will show perceptual asymmetries when presented with non-native consonants that make use of finer place contrasts within one of the major places.

The characteristics of Australian languages provide an optimal test-case for these predictions. Many have three or four-way within-coronal place contrasts in stops (and nasals and laterals). Commonly, some of these contrasts are neutralised, or are unevenly distributed, in one or more positions in words, creating the conditions for stark differences in the distributional frequency among the individual coronal consonants. We turn next to a brief description of our target langauge, Wubuy, with particular focus on its phonological and phonetic characteristics.

### 1.4 Wubuy

Wubuy, also known as ‘Nunggubuyu’ [11], is an indigenous Australian language spoken in south-eastern Arnhem Land, traditionally around the southern part of Blue Mud Bay in the Gulf of Carpentaria and now mostly in the settlement of Numbulwar. It is the first language for adults over the age of around 55 in the community, as well as a first or second language for many adults of the same generation in neighbouring Groote Eylandt. Wubuy is a critically endangered langauge—children are no longer acquiring it as a first language, though all children in the community are exposed to some Wubuy, both through their grandparents and through local language revitalisation efforts at the school. There are perhaps 60 first language speakers of Wubuy in Numbulwar.

The consonantal inventory of Wubuy is presented in Table 1. As can be seen from an examination of this table, the phonology of Wubuy has a four-way place distinction among the coronal stops /t ʈ $\underset{̪}{\text{t}}$ ȶ/ (apico-alveolar, apico-retroflex, lamino-dental, lamino-alveopalatal). The contrast is found word-initially as well as medially, as noted above. This is unusual for languages with multiple coronal stop contrasts. A survey of the phonotactics of Australian languages presented in [10] identifies just 10 languages (from 116) where the four coronal stops are contrastive initially, as well as medially; most of these languages are now, however, extinct or moribund. Despite being endangered, Wubuy is among the 50 or so Australian languages that still have a sizeable speech community of more than 5–10 speakers [40] and, of those, one of the very few with an initial four-way contrast.

**Table 1:** The phonemic consonant inventory of Wubuy, adapted from [11].

	Labial	Lamino-dental	Apico-alveolar	Apico-post-alveolar (retroflex)	Lamino-alveopalatal	Dorso-velar
**Stop**	p	$\underset{̪}{\text{t}}$	t	ʈ	ȶ	k
**Nasal**	m	$\underset{̪}{\text{n}}$	n	ɳ	ȵ	ŋ
**Lateral**		$\underset{̪}{\text{l}}$	l	ɭ		
**Tap/trill**			r			
**Approx.**	w			ɻ	j	

Previous acoustic work on Wubuy [41, 42] shows that there are very small acoustic differences among three of the coronal stops: the apico-alveolar /t/, the apico-retroflex /ʈ/, and the lamino-dental /$\underset{̪}{\text{t}}$/. We refer to this group, following [43], as the [—sharp] set of coronals. We justify this grouping further in section 4.1.1. The great degree of acoustic and articulatory overlap among the 3 [—sharp] coronals makes Wubuy ideal for testing the limitations of native segmental perception in the absence of lexical information.

Given suggestions in the literature that 'listeners are biased towards accepting a frequent pattern more often than an infrequent one' [44], we examined the distributional properties of coronal stops in Wubuy. Indeed, the distributional frequencies of the coronal stops in spoken Wubuy differ systematically across the four places of articulation in ways that provide opportunity for evaluating the influence of input frequency on overriding innate perceptual asymmetries among coronal stops, discussed in [45]. A phoneme count of 8% of the Wubuy text corpus [46] (i.e., six of the texts), shows that the lamino-alveopalatal stop is far more frequent than the dental, alveolar and retroflex stops, except in word-initial position where the alveolar is somewhat more frequent (we use 'word' here in the ordinary syntactic sense of 'terminal elements in phrase structure'). The lamino-alveopalatal is also much more frequent than the other coronal stops in initial position in verb stems (see Table 2), which typically does not correspond to word-initial position because Wubuy is a language with obligatory inflectional prefixes (see, for instance, [47, 48, 49, 50] for discussion of the cognitive saliency of stems in prefixed words). The retroflex is infrequently represented, both overall and also in stem-initial position in both verbs and nouns. This is what distinguishes the retroflex from the dental, which, although infrequent word-initially, is equal to the alveolar in the highly salient verb stem-initial position in which both are twice as frequent as the retroflex. Thus, combining the two most prominent phonotactic and morphological positions—word-initial and verb stem-initial—the order of frequency of occurrence of the coronal stops overall is: ȶ > t, $\underset{̪}{\text{t}}$ > ʈ. Wubuy thus provides not only an optimal stop consonant inventory for the testing of the basic assumptions of an NRC hypothesis, it also provides differences in the distributional frequency of those native stop consonants in the spoken input, which may affect the status of these segments in the minds of speakers.

**Table 2:** Frequencies of stop consonant phonemes in all words in 8% of the Wubuy text corpus [46], adapted from [45]. The ‘Initial’ column presents the word-initial distribution of stops in the words in the Wubuy texts, while the ‘Total’ column indicates the distribution of stop phonemes in all word positions. The ‘Verb Initial’ column indicates the distribution of stop consonants in verb stem-initial position.

				Word-		Verb-	
	Phoneme	Total	%	Initial	%	Initial	%
**Stops**	**p**	561	6.21	64	3.88	344	8.68
	**t**	175	1.94	31	1.88	67	1.69
	**ʈ**	118	1.31	11	0.67	33	0.83
	$\underset{̪}{t}$	130	1.44	11	0.67	67	1.69
	**ȶ**	464	5.13	24	1.45	123	3.1
	k	835	9.24	40	2.42	125	3.15
**Total**		**2283**	**25.27**	**181**	**10.97**	**759**	**19.14**

### 1.5 Predictions

In the following, we present two two-part studies of perception of the acoustically and articulatorily similar Wubuy coronal stops /t ʈ $\underset{̪}{\text{t}}$ ȶ/. In study 1a and 1b, we tested native speakers of Wubuy and, in study 2a and 2b, we tested native speakers of Australian English as listeners who are naïve to this contrast series. Studies 1a and 2a presented the consonant targets in word-medial position (/aCa/). Studies 1b and 2b tested discrimination of the three [—sharp] stops in absolute initial position (/##Ca/).

In the case of the Wubuy listeners, we test the following competing hypotheses:

**H1) All phoneme categories are distinct entities in the phonological grammar of a listener, and native language learning ensures that all native phonemes are perceived in equally categorical fashion.** In the light of the literature reviewed above, however, this general hypothesis is considered unlikely to be supported.

**H2) Native phoneme discrimination performance is subject to factors such as acoustic and/or articulatory similarity/overlap among native phonemes, as well as by frequency and distribution in the input.** This hypothesis is compatible with PAM/PAM-L2 in conjunction with the NRC hypothesis, as argued above. For the present studies, this hypothesis would predict that:

**H2a: acoustic/articulatory distinctiveness)** Acoustically and articulatorily well-differentiated contrasts, i.e., /ȶ t/, /ȶ t/$\underset{̪}{}$ and /ȶ ʈ/, will be more successfully discriminated even by non-native listeners than those involving contrasts between [—sharp] coronals, as the former three contrasts will likely result in a typical TC discrimination pattern in PAM/PAM-L2 terminology.

**H2b: acoustic/articulatory overlap)** In contrast, the [—sharp] stops are more likely to be confused with each other and result in poorer, though still above chance, native discrimination scores. The discrimination accuracy will depend on the specific acoustic/articulatory similarities and differences of each pair, and also by the consonants’ frequency and distribution in spoken Wubuy (because over-riding the bias towards the canonical articulations requires sufficient input: see H2c). We predict good discrimination of /$\underset{̪}{\text{t}}$ ʈ/ as these are the most articulatorily and acoustically distinct of the contrasts among [—sharp] coronal stops ([43]; and c.f., [42]).

**H2c: effects of frequency in the native input)** Despite the claim that /$\underset{̪}{\text{t}}$ ʈ/ are the most distinct pair of [—sharp] coronal stops, we nevertheless expect that discrimination of /t $\underset{̪}{\text{t}}$/ will be better than both /$\underset{̪}{\text{t}}$ ʈ/ and /t ʈ/, because of their differing lexical frequencies ([t, $\underset{̪}{\text{t}}$] > ʈ). Specifically, we expect /$\underset{̪}{\text{t}}$ ʈ/ and /t ʈ/ to exhibit asymmetries in discrimination, akin to those reported for vowels [12, 13, 14, 15]. Indeed, we predict that both the alveolar and the dental stops will act as perceptual anchors, due to their greater frequency in the input, and in the case of the alveolar, due to it being the 'canonical' coronal stop. Moreover, because of this we expect the relative performance levels on these contrasts to most clearly differentiate the native Wubuy listeners and Wubuy-naïve English listeners.

**H2d: effect of context**) Finally, we predict that Wubuy speakers will perform similarly in the two contexts examined here: /aCa/ and /##Ca/, with the exception of the /t ʈ/ contrast, which they should have more difficulty with in initial than in medial position as /ʈ/ is extremely rare in initial position (and also because of claims in the literature that /ʈ/ is difficult to distinguish from /t/ in the absence of a preceding vowel [9, 10, 22]).

In the case of the Wubuy-naïve English listeners, we test the following hypotheses:

**H3)** English listeners will perceive /ȶ/ as an instance of English /tʃ/ (or /dʒ/), while the [—sharp] stops will all be perceived as English /t/ (or /d/): /t/ will be perceived as a perfectly good /t/, while both /$\underset{̪}{\text{t}}$/ and /ʈ/ will be perceived as (somewhat) ‘odd’ /t/s. Contrasts involving the lamino-alveopalatal will thus become TC assimilations in PAM/PAM-L2 terminology, and discrimination accuracy is expected to be excellent. The [—sharp] contrasts will be perceived as CG contrasts, and discrimination will be poor to moderately good, depending on the magnitude of the acoustic and articulatory difference between the stops in each contrast. This is consistent with the report that native speakers of English struggle to discriminate English (alveolar, short-lag voiced) /d/ and French (dental, prevoiced) /d/, presumably because they perceive both the native and non-native /d/ as instances of the same native phonological category [51].

**H4)** On the basis of the NRC hypothesis, we further predict that order of presentation will be important for the two most acoustically/articulatorily similar coronal contrasts, /$\underset{̪}{\text{t}}$ t/ and /ʈ t/ such that it will be easier to discriminate these contrasts when the 'odd' (and non-canonical) consonant is presented first.

**H5)** Finally, we expect English listeners to perform equally well in the two positional contexts, as they have no previous experiences with multiple coronal stops in either context.

## Method

This research including the consent procedure was approved by the University of Melbourne’s Human Research Ethics Committee [1035119]. All participants provided written consent. For the Wubuy speakers, all consent was obtained by RB-N and BB in the presence of a native Wubuy speaker who acted as an interpreter when necessary.

### 2.1 Stimuli

We recorded three female native speakers of Wubuy (ages 51–61 years), born and raised in the Numbulwar area by native speakers of Wubuy. Two participants also reported speaking the neighbouring Aboriginal language Enindhilyakwa (Groote Eylandt) with relatives (grandparents, in-laws), and all understand and speak the community language Roper Kriol (an English-lexified creole: [52]) to some extent. All three speakers had acquired English as a second language in a classroom setting. All had basic linguistic training.

Each of the three participants produced the four target consonants /t ʈ $\underset{̪}{\text{t}}$ ȶ/ in the two contexts given in (1), except for the lamino-alveopalatal /ȶ/ which was only produced in the intervocalic /aCa/ context. The target word list is shown in Table 3. Note that the primary stress is on the first syllable of each root word.

(1) Context 1 Phrase-medial, word-internal /aCa/ targets
  Context 2 Utterance initial, word-initial /##Ca/ targets

**Table 3:** Target words for each context. Note that the orthographic representation (leftmost column for each context) represents the alveolar stop by ‘d’, the retroflex by underscoring ‘d’, and the dental by ‘dh.’ The other columns provide a phonetic transcription of the standard Wubuy pronunciation of the word in (IPA), and its English gloss.

**Wordlist for the /aCa/ context**		
*Orthography*	*IPA*	*Gloss*
*maada*	[ˈmaːta]	‘pipe’
*ma* *d* *a*	[ˈmaʈa]	‘grass’
*madhal*	[ˈmat$\underset{̪}{\text{t}}$al]	‘leech’
*maja*	[ˈmaȶa]	'seagrass'
**Wordlist for the /##Ca/ context**		
*Orthography*	*IPA*	*Gloss*
*dawal*	[ˈtawal]	‘axe shaft junction’
*d* *angga* *l* *garra*	[ˈʈaŋkaɭˌkaɾa]	‘lancewood’
*dhawal*	[ˈ$\underset{̪}{\text{t}}$awal]	‘coccyx’

The target words were selected to provide a symmetrical vowel context on either side of the consonants in the /aCa/ context. The Wubuy carrier phrases were chosen so as to minimize coarticulation with the adjacent edges of the carrier phrase. The carrier phrase for the word-medial elicitations is given in (2a) in both Wubuy orthography and IPA. The carrier phrase for the utterance-initial elicitations, in (2b), is a re-ordering of the same words. Wubuy syntax is non-configurational [11], and both carrier phrases are acceptable to native speakers.

(2) a. ‘*nga-yamana __________adaba*’    b. ‘*__________ nga-yamana a* *d* *aba*’

          [ˈŋa-jamana ________ˈaʈapa]            [ __________ ˈŋa-jamana    ˈaʈapa]

          1SG-say.PRS                now                                       1SG-say.PRS    now

          ‘I say __________        now’                 ‘__________, I say now’

### 2.2 Recording procedure and stimulus treatment

The three speakers read the target words in the carrier sentences presented in Wubuy orthography on a computer monitor in a fixed order, blocked by the type of consonant. The participants were encouraged to discuss and rehearse the words prior to the recording to ensure recognition of all target items during the recording. The participants were instructed to speak in a clear, comfortable voice as though they were speaking to a friend. Five correct utterances (as judged by the speaker herself, as well as by the other speakers who were present in the room during the recording, though they remained quiet throughout, only motioning silently with face/head and hands) were recorded for each target, resulting in a total of 45 correct utterances (5 tokens per 4 targets in the /aCa/ context, and 5 tokens per 3 targets in the /##Ca/ context). Recordings of targets containing coughs, stutters or speech or reading errors were discarded/replaced.

For the recording, we used a Shure SM10A headset cardioid microphone, an EDIROL UA-25 USB audio interface, and a laptop computer with Cool Edit 2000. All recordings had a 16-bit sampling depth with a sampling rate of 44.1 KHz. The recordings were made in a sound-attenuated professional recording studio at MARCS Institute in Sydney. The target /aCa/ and /##Ca/ sequences were excised using a *Praat* script and checked by the first and second author. Each excised token was given a 20 millisecond ramp-in and a 10 millisecond ramp-out using *Praat*.

### 2.3 Stimulus presentation

The excised /aCa/ and /##Ca/ targets were presented to our participants in the form of two separate randomised cross-speaker categorical XAB discrimination tasks (for both the /aCa/ and /##Ca/ contexts) programmed in *Psyscope X*, with the stimuli presented over headphones from a MacBook laptop computer. A cross-speaker discrimination task presents the listener with speech tokens from three different speakers in each XAB triad, and thus forces the listeners to disregard differences in voice quality and other idiosyncracies, and instead to focus exclusively on the phonological information in order to complete the task successfully.

The XAB discrimination task was explained to the participants as a task in which a ‘teacher’ (the first voice heard) was being imitated by a ‘good student’ and a ‘bad student’ (voices 2 and 3), and it was the job of the participant to indicate (with a key press on the keyboard) which of the two students (voice 2 or 3) was the ‘good student’ who copied correctly what the teacher had said. We provided this explanation to make the experimental paradigm meaningful to our participants and increase the likelihood that they would understand the task at hand and be able to complete the experiments. Similar stories and explanations are often provided in perception research, especially to children and other participants who are unfamiliar with experimental research.

For both studies, the inter-stimulus interval (ISI) between stimuli within each XAB trial was 500 ms. The response window was presented for two seconds following the playing of the third target. If the participant did not respond within the two-second window, the trial was replayed later. The inter-trial interval was one second.

In both studies, the participants were presented with six unique triads and six repetitions per contrast type, equaling a total of 36 triads per contrast for each listener. There were a total of 12 contrasts (all combinations of the four consonants /t ʈ $\underset{̪}{\text{t}}$ ȶ/ as there might be differences in discriminability depending on the order of presentation) in the /aCa/ context (432 trials total per listener) and six contrasts (all combinations of the three consonants /t ʈ $\underset{̪}{\text{t}}$/, again allowing for differences in discriminability due to order of presentation) in the /##Ca/ context (216 trials total per listener). The participants in each study first completed the discrimination of the target consonants in medial position (the /aCa/ context (Study 1a, 2a), and then the discrimination of the target consonants in initial position (the /Ca/ context (Study 1b, 2b).

### 2.3 Participants

#### Wubuy participants

The participants were 10 native speakers of Wubuy (age 40–65 approximately; one male). Some were literate and some semi-literate in Wubuy. In addition to Wubuy, the participants spoke (and read) English and the community language Roper Kriol to varying levels of proficiency, similarly to the recorded speakers. Another six Wubuy speakers were tested, but were excluded from the analysis: four failed to understand the task, one was reluctant to complete the task, and one reported that her first language was not Wubuy. The number of subjects tested therefore was around 25% of the total estimated speaker population of 60.

All testing of Wubuy listeners took place in a sound-attenuated booth at Numbulwar School, in Numbulwar, NT. All procedures were explained to the participants in English or Kriol by the first and second authors and in Wubuy by a native speaker who assisted with interpretation and translation when needed. Each participant was compensated by a $100 payment.

#### Australian English participants

The participants were 11 native speakers of English (age 18–53, *M* = 25.6 years: four male). All were university students, recruited by word of mouth. Approximately half were undergraduate linguistics students with some knowledge of phonology and phonetics. All were native speakers of Australian English, though nine reported having some knowledge of other languages acquired through formal language instruction at school or university. None of the languages studied by the participants contrast multiple coronal stops based on place of articulation. One additional participant was tested but excluded due to self-reported hearing loss. Another was excluded as she had non-Australian English-speaking parents.

All testing of the Australian English listeners took place at the Department of Linguistics at University of Melbourne or at MARCS Institute, University of Western Sydney. All procedures were explained to the participants by the first and second authors. Each participant was compensated by a $20 payment.

## Results

For all statistical inference reported in the following, we used non-parametric tests. Since the data consist of ratios (percentages) and are not always normally distributed, standard non-parametric equivalents to *t*-tests and ANOVA were employed, using the statistics software SPSS. These tests were:

1. One-sample Wilcoxon signed-rank tests–to test whether the sample median is different from a hypothesised value. In this paper the hypothesised value is always the value expected due to chance performance.
2. Related-samples Wilcoxon signed-rank tests–to test whether the sample medians of two related samples are different. In the paper, it is primarily applied to evaluate context differences.
3. Related-samples Friedman's analysis of variance by ranks–to test whether the distributions of several samples differ from each other. The Friedman test [53] is a non-parametric alternative to one-way repeated-measures ANOVA. Posthoc comparisons are computed and the resulting p-values adjusted for multiple comparisons using the Dunn-Bonferroni test procedure [54].
4. Independent-samples Mann-Whitney *U* tests–to test whether the sample median of two independent samples are different. In the paper, it is applied when comparing the results from the two different listener groups.

### 3.1 Native perception

**Fig 1:**
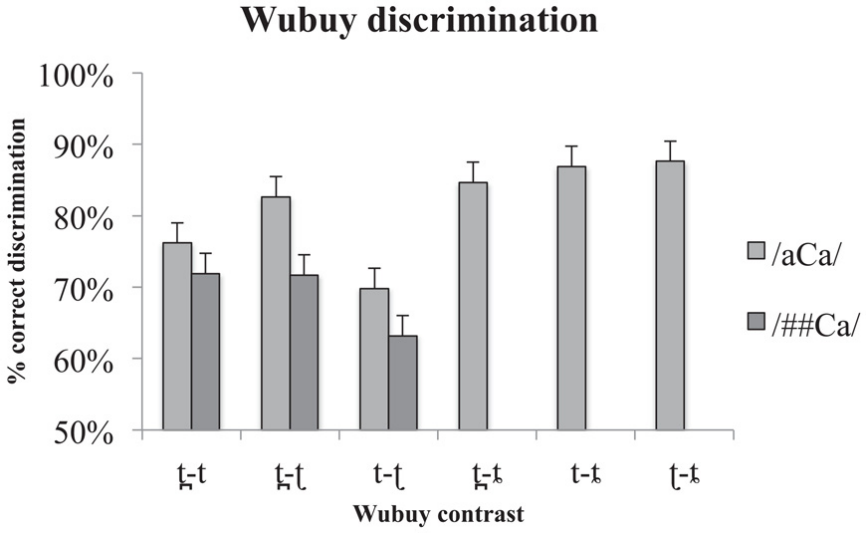
Native discrimination scores per contrast type in the /aCa/ and /##Ca/ contexts. % correct discrimination is presented on the y-axis, while contrast type is presented on the x-axis. Error bars indicate standard error of the mean. Note, however, that as the statistics applied are non-parametric, they are not necessarily related to the dispersion indicated in the error bars.

The results of the /aCa/ discrimination task are presented in Fig 1. Chance performance is 50% correct discrimination. As is evident from Fig 1, the Wubuy speakers were indeed able to discriminate the four coronal stops in their native language (confirmed using one-sample Wilcoxon signed rank tests against chance performance with *p* values < .05 for all contrasts). The mean discriminability of the six contrasts in the medial context was 81% correct. One-sample Wilcoxon signed rank tests against chance performance also confirmed that the Wubuy speakers were able to discriminate the three [—sharp] contrasts tested in the /##Ca/ context (see Fig 1), with *p* values < .05 for each contrast. The mean discriminability of the three contrasts in the /##Ca/ context was 69% (range 63% correct for /$\underset{̪}{\text{t}}$ ʈ/ to 72% correct discrimination for /t $\underset{̪}{\text{t}}$). This suggests that discrimination is more difficult in initial position and a related-samples Wilcoxon signed rank test of the distribution of scores in the /aCa/ and /##Ca/ contexts confirmed this with a significant effect of context (*p* = .037). There was no significant effect of contrast, which is unsurprising as we did not predict an overall loss or gain in discriminability.

However, as laid out in H2b and H2c above, we predicted that /t ʈ/ would be less accurately discriminated than /$\underset{̪}{\text{t}}$ ʈ/ and /t $\underset{̪}{\text{t}}$/, due in the case of /$\underset{̪}{\text{t}}$ ʈ/ to maximal acoustic differentiation, and in the case of /t $\underset{̪}{\text{t}}$/ to the fact that /t/ and /$\underset{̪}{\text{t}}$/ are more frequent in Wubuy input than /ʈ/. A related-samples Friedman's analysis of variance by ranks comparing the distribution of the averaged scores in the two contexts indicated that there was indeed an effect of contrast (*p* = .009), and Dunn-Bonferroni post-hoc comparison of the differences between the discrimination accuracy for the three averaged contrasts confirmed that the /t ʈ/ contrast was indeed discriminated less accurately than the /$\underset{̪}{\text{t}}$ ʈ/ as predicted. The /t ʈ/ discrimination accuracy did not however differ significantly from that of /t $\underset{̪}{\text{t}}$/. We suspect that this is due to the poor acoustic differentiation of /t $\underset{̪}{\text{t}}$/ offering little information for the listeners on which to base their discrimination [42]. In terms of an effect of context on the discrimination accuracy of individual contrasts, we expected that the discrimination accuracy of the acoustically similar /t ʈ/ would decrease from the /aCa/ context to the /##Ca/ context due to the infrequency of /ʈ/ in initial position in Wubuy input (H2d). A related-samples Wilcoxon match-pair signed-rank test did not support our hypothesis (*p* = .33), though the difference between the two contexts is in the predicted direction.

Interestingly, however, a related-samples Friedman's analysis of variance by ranks of each condition (/aCa/ and /##Ca/) separately showed that the discrimination performance of the participants varied with the contrast in question in the /aCa/ context, with correct discrimination ranging from 88% correct for /ʈ ȶ/, to as low as 70% for /t ʈ/ contrast (medial position *p* = .045; initial position *p* = .139, *ns*).

Subsequent Dunn-Bonferroni post-hoc comparison of the differences in the /aCa/ context showed that the only significant difference was in the discrimination accuracy of the apical contrast /t ʈ/ which was less accurate than discrimination of either of the apicals with the lamino-alveopalatal. This clearly suggests that the apical contrast is the most difficult to discriminate even for native speakers, and even in medial position where it is better supported by acoustic cues on both sides, i.e., during consonant closure as well as release.

### 3.2 Non-native perception

**Fig 2:**
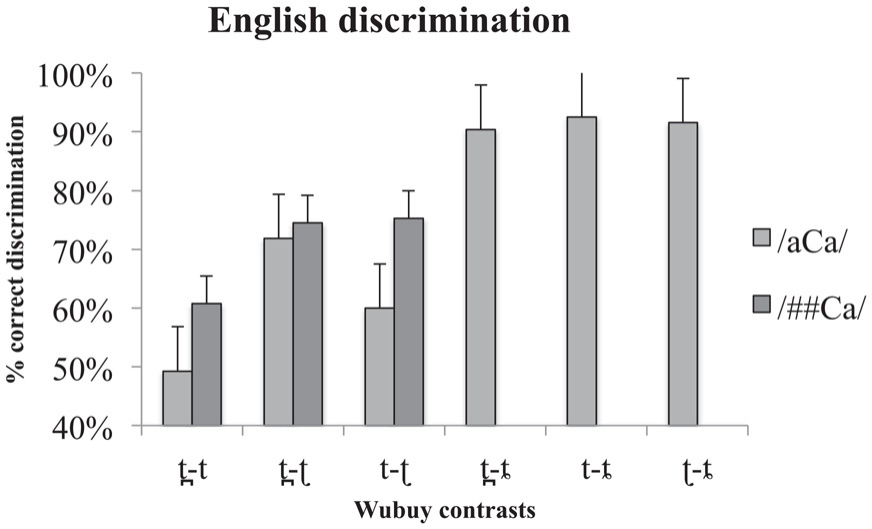
Non-native discrimination scores per contrast type in the /aCa/ and /##Ca/ contexts. % correct discrimination is presented on the y-axis, while contrast type is presented on the x-axis. Error bars indicate standard error of the mean. Note, however, that as the statistics applied are non-parametric, they are not necessarily related to the dispersion indicated in the error bars.

The results for the non-native English listeners’ /aCa/ discrimination are presented in Fig 2. As before, chance performance is 50% correct discrimination. As the figure indicates, the English listeners were able to discriminate most of the coronal stops in Wubuy (% correct discrimination ranged from non-significant discrimination of 49% for /$\underset{̪}{\text{t}}$ t/ to 93% for /t ȶ/, with all three contrasts involving /ȶ/ being discriminated correctly in more than 90% of cases). One-sample Wilcoxon signed rank tests against chance performance confirmed that the English listeners were indeed able to discriminate the Wubuy stops (*p* = < 0.05 for /t ʈ/, /ʈ $\underset{̪}{\text{t}}$/, /t ȶ/, /$\underset{̪}{\text{t}}$ ȶ/, and /ʈ ȶ/), with the exception of /$\underset{̪}{\text{t}}$ t/ which was not significant in the /aCa/ condition. One-sample Wilcoxon signed rank tests against chance performance also confirmed that the English listeners performed above chance (*p* < 0.05 for all three contrasts) in the /##Ca/ condition. Note that the discrimination performance for /$\underset{̪}{\text{t}}$ t/, which had *not* been discriminated in /aCa/ context, was above chance in /##Ca/ context.

As in the case of the Wubuy listeners, a related-samples Wilcoxon signed rank test of the distribution of scores in the /aCa/ and /##Ca/ contexts showed a significant main effect of context (*p* = .013), which we did not predict. However, as opposed to the Wubuy listeners, the English listeners found /##Ca/ discrimination to be easier than the /aCa/ (confirmed by an independent samples Mann-Whitney U test, *p* < .001). This likely reflects an increased familiarity with the Wubuy stops (and perhaps the task) as the /##Ca/ study was always presented second. As predicted, there was no significant effect of contrast.

A related-samples Friedman's analysis of variance by ranks showed that there was a significant effect of contrast type in both conditions separately (/aCa/ *p* = .000; /##Ca/ *p* = .006). Subsequent Dunn-Bonferroni post-hoc comparisons showed that, in the /aCa/ context the /t $\underset{̪}{\text{t}}$/ contrast and the apical contrast /t ʈ/ were less accurately discriminated than all contrasts involving a lamino-alveopalatal (all significant at or below *p* = .005). Post-hoc comparisons of the results in the /##Ca/ context showed that the /t $\underset{̪}{\text{t}}$/ contrast was significantly more poorly discriminated than /$\underset{̪}{\text{t}}$ ʈ/ (*p* = .012) and /t ʈ/ (*p* = .032). There was no significant difference in the discriminability of the apical contrast /t ʈ/ and the dental-retroflex contrast /$\underset{̪}{\text{t}}$ ʈ/.

### 3.3 Native versus non-native perception

**Fig 3:**
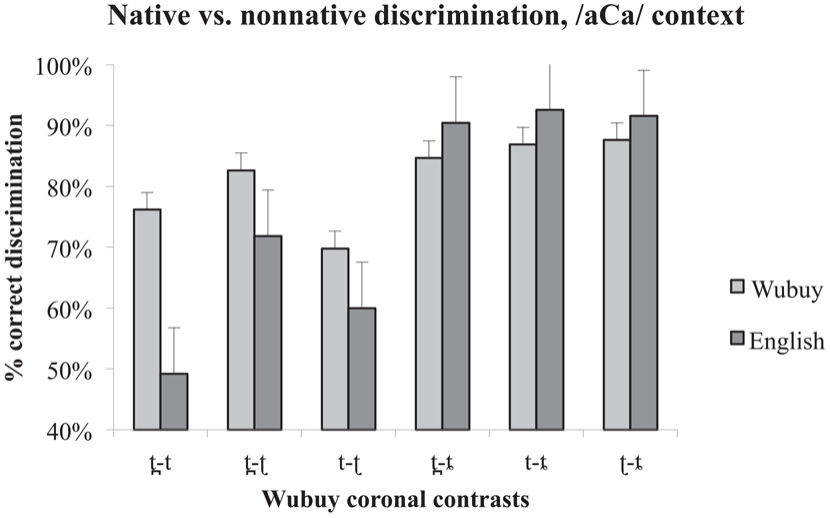
Comparison of native and non-native discrimination performance in the /aCa/ context. % correct discrimination is presented on the y-axis, while contrast type is presented on the x-axis. Error bars indicate standard error of the mean. Note, however, that as the statistics applied are non-parametric, they are not necessarily related to the dispersion indicated in the error bars.

**Fig 4:**
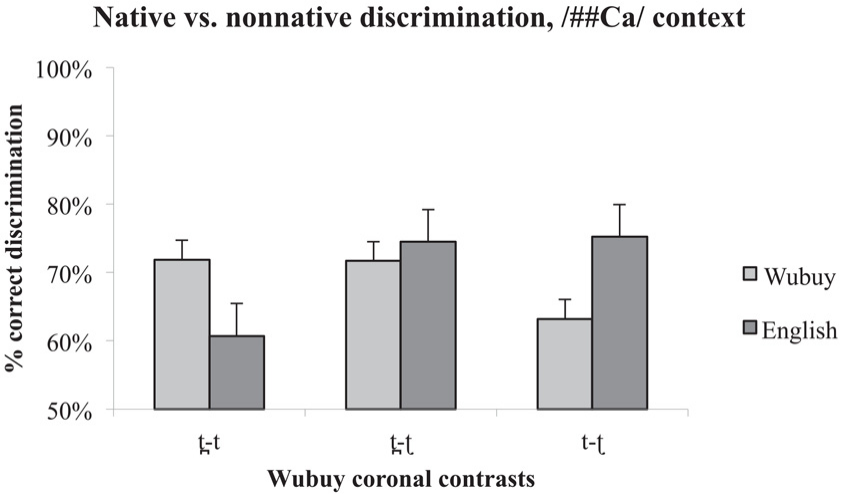
Comparison of native and non-native discrimination performance in the /##Ca/ context. % correct discrimination is presented on the y-axis, while contrast type is presented on the x-axis. Error bars indicate standard error of the mean. Note, however, that as the statistics applied are non-parametric, they are not necessarily related to the dispersion indicated in the error bars.

In order to assess what similarities and/or differences exist in the performance of the two groups with regard to each of the contrasts in each presentation environment (see Fig 3 for the /aCa/ context, and Fig 4 for the /##Ca/ context), we conducted six independent-samples Mann-Whitney U tests. Table 4 shows that the two listener groups differed in their discrimination of medial /t $\underset{̪}{\text{t}}$/ (where the Wubuy listeners out-performed the English listeners), and in their discrimination of initial /t ʈ/ (where the English listeners out-performed the Wubuy listeners).

**Table 4:** Pairwise comparison, Wubuy versus English discrimination in /aCa/ and /##Ca/, excluding contrasts involving the lamino-alveopalatal. Significant results indicated by * and Sig. in bold.

	Contrast	Mean Group Difference	Sig
	t $\underset{̪}{\text{t}}$	-.269*	**0.001**
**/aCa/**	t ʈ	-0.098	0.173
	ʈ $\underset{̪}{\text{t}}$	-0.108	0.099
	t $\underset{̪}{\text{t}}$	-0.111	0.085
**/##Ca/**	t ʈ	.121*	**0.013**
	ʈ $\underset{̪}{\text{t}}$	0.028	0.705

### 3.4 Discrimination asymmetries in native and non-native listeners

In order to test the Natural Referent Consonant hypothesis outlined in the introduction, and its predictions in H2c and H4 that discrimination accuracy may differ in ways specific to each listener group depending on the order of presentation, we conducted one further set of analyses of our discrimination results for the contrasts /t ʈ/, /$\underset{̪}{\text{t}}$ ʈ/ and /t $\underset{̪}{\text{t}}$/. As our participants were presented with a well-balanced stimulus set, where all consonants appeared first and in combination with all other consonants, we were able to split the discrimination results from each contrast into two separate sub-analyses for each contrast: one in which each member of the contrast was presented first, and one where it was presented second. The sub-divided results are presented in Table 5 (Wubuy) and Table 6 (English) below.

**Table 5:** Wubuy discrimination accuracy depending on the first presented consonant for /t ʈ/, /$\underset{̪}{\text{t}}$ ʈ/ and /t $\underset{̪}{\text{t}}$/ in both the /aCa/ (first mean % listed) and /##Ca/ (second mean %) contexts.

Initial		Accuracy		Initial		Accuracy
consonant	Triad	aCa/##Ca		consonant	Triad	aCa/##Ca
**t**	t t ʈ	57%/55%	vs	**ʈ**	ʈ ʈ t	77%/65%
	t ʈ t				ʈ t ʈ	
$\underset{̪}{t}$	$\underset{̪}{\text{t}}\;\underset{̪}{\text{t}}$ ʈ	57%/67%	vs	**ʈ**	ʈ ʈ $\underset{̪}{\text{t}}$	77%/84%
	$\underset{̪}{\text{t}}$ ʈ $\underset{̪}{\text{t}}$				ʈ $\underset{̪}{\text{t}}$ ʈ	
**t**	t t $\underset{̪}{\text{t}}$	68%/64%	vs	$\underset{̪}{t}$	$\underset{̪}{\text{t}}\;\underset{̪}{\text{t}}$ t	67%/62%
	t $\underset{̪}{\text{t}}$ t				$\underset{̪}{\text{t}}$ t $\underset{̪}{\text{t}}$	

**Table 6:** English discrimination accuracy depending on the first presented consonant for /t ʈ/, /$\underset{̪}{\text{t}}$ ʈ/ and /t $\underset{̪}{\text{t}}$/ in both the /aCa/ and /##Ca/ contexts.

Initial		Accuracy		Initial		Accuracy
consonant	Triad	aCa/##Ca		consonant	Triad	aCa/##Ca
**t**	t t ʈ	57%/73%	vs	**ʈ**	ʈ ʈ t	61%/78%
	t ʈ t				ʈ t ʈ	
$\underset{̪}{t}$	$\underset{̪}{\text{t}}\;\underset{̪}{\text{t}}$ ʈ	70%/71%	vs	**ʈ**	ʈ ʈ $\underset{̪}{\text{t}}$	70%/78%
	$\underset{̪}{\text{t}}$ ʈ $\underset{̪}{\text{t}}$				ʈ $\underset{̪}{\text{t}}$ ʈ	
**t**	t t $\underset{̪}{\text{t}}$	39%/59%	vs	$\underset{̪}{t}$	$\underset{̪}{\text{t}}\;\underset{̪}{\text{t}}$ t	58%/67%
	t $\underset{̪}{\text{t}}$ t				$\underset{̪}{\text{t}}$ t $\underset{̪}{\text{t}}$	

If our NRC hypothesis predictions are correct, we expect the following patterns:

1. Wubuy listeners will show an asymmetry in discriminating the infrequent /ʈ/ from both /t/ and /$\underset{̪}{\text{t}}$/, such that discrimination will be more accurate when the infrequent consonant is presented first (and thus twice) in each triad (H2c).
2. Wubuy listeners will display no asymmetry when they are discriminating the two frequently-occurring coronals /$\underset{̪}{\text{t}}$/ and /t/ (H2c).
3. English listeners will show an asymmetry between /t $\underset{̪}{\text{t}}$/ and /t ʈ/ such that discrimination is easier when the ‘odd’ consonant relative to English (/$\underset{̪}{\text{t}}$/ or /ʈ/) rather than the native (and more canonical) /t/ is presented first. They are not, unlike Wubuy listeners, expected to show any asymmetry for the contrast /$\underset{̪}{\text{t}}$ ʈ/, because both phones are 'odd' in this case (H4). We qualify this with the fact that we are not basing our predictions on a categorisation task, as we have not carried out such a study. It is possible that the English participants’ perception of the Wubuy stops differs from what we are proposing.

**Fig 5:**
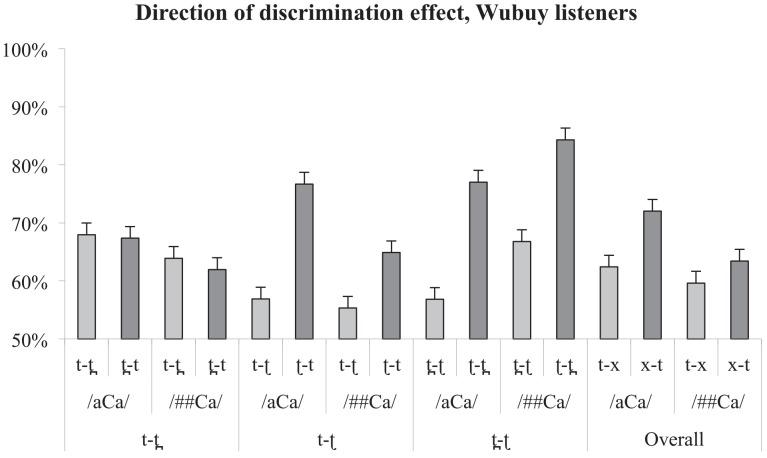
Discrimination asymmetries for Wubuy listeners. Error bars indicate standard error of the mean. Note, however, that as the statistics applied are non-parametric, they are not necessarily related to the dispersion indicated in the error bars.

**Fig 6:**
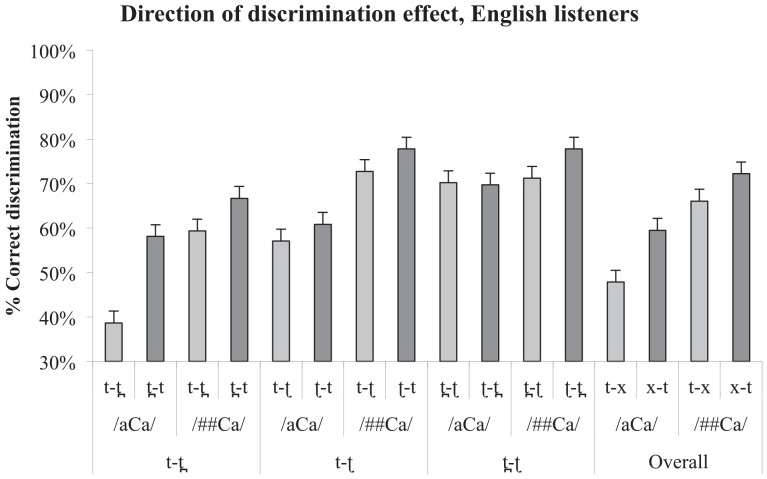
Discrimination accuracy for English listeners. Error bars indicate standard error of the mean. Note, however, that as the statistics applied are non-parametric, they are not necessarily related to the dispersion indicated in the error bars.

The discrimination asymmetries for the three contrasts (and an additional measure of /t/ versus a collapsed category 'x' which is an average of the /$\underset{̪}{\text{t}}$/ and /ʈ/). are presented in Figs 5 and 6, below. Asymmetry values were computed by subtracting the paired percentage correct values for the two presentation orders from each other.

We tested for an overall effect of presentation order for native and non-native listeners separately and found a significant effect in both listener groups (asymmetry values averaged over contrast and context and submitted to Wilcoxon signed-rank test against the hypothesised median value of 0; Wubuy listeners: *p* = .005; English listeners: *p* = .018). We then subjected the individual results for each context-contrast cell to a series of One-sample Wilcoxon signed-rank tests to determine whether there were any differences, for each group, between the performance on each contrast pair according to direction of presentation. We also compared the two groups for the difference in their performance on contrast-by-presentation order. Neither of these results differed significantly from chance. As the descriptive statistics make clear, this is probably due to the unavoidably low number of participants (approximately 25% of the entire Wubuy speech community) resulting in a loss of statistical power, and the great degree of variance in both groups, for all contrasts. Indeed, the only result which reached statistical significance (and this on a one-sample *t*-test against 0) was that of the contrast /$\underset{̪}{\text{t}}$ ʈ/ between English and Wubuy listeners, which is the largest difference in means in the entire sample. We discuss this issue further in Section 4.

Nevertheless, the differences in the means alone (shown in Tables 5 and 6) are consistent with a difference between contrasts according to presentation order, and according to group, as predicted. We therefore subjected the results to further one-sample Wilcoxon signed rank tests against the hypothetical value of 0 (no difference between presentation orders). *P*-values are presented in Tables 7 and 8. As is clear, the Wubuy listeners show the expected direction of perceptual asymmetry for the contrasts /t ʈ/ and /$\underset{̪}{\text{t}}$ ʈ/, consistent with the low frequency of occurrence of /ʈ/ in the speech input.

**Table 7:** Results of one-sample Wilcoxon signed rank tests of the perceptual asymmetries of Wubuy listeners. Significant results in bold.

Context	Contrast	Mean %	*Mean diff*.	Sig.
**/aCa/**	**t** $\underset{̪}{t}$	68	0.6	ns
	$\underset{̪}{t}$ **t**	67		
	**t ʈ**	57	-19.8	**0.03**
	**ʈ t**	77		
	$\underset{̪}{t}$ **ʈ**	57	-20	**0.036**
	**ʈ** $\underset{̪}{t}$	77		
**/##Ca/**	**t** $\underset{̪}{t}$	64	1.9	ns
	$\underset{̪}{t}$ **t**	62		
	**t ʈ**	55	-9.6	**0.011**
	**ʈ t**	65		
	$\underset{̪}{t}$ **ʈ**	67	-17.5	**0.038**
	**ʈ** $\underset{̪}{t}$	84		

**Table 8:** Results of one-sample Wilcoxon signed rank tests of the perceptual asymmetries of English listeners. Significant results in bold.

Context	Contrast	Mean %	*Mean diff*.	Sig.
**/aCa/**	**t** $\underset{̪}{t}$	39	-19.4	**0.022**
	$\underset{̪}{t}$ **t**	58		
	**t ʈ**	57	-3.8	ns
	**ʈ t**	61		
	$\underset{̪}{t}$ **ʈ**	70	0.5	ns
	**ʈ** $\underset{̪}{t}$	70		
**/##Ca/**	**t** $\underset{̪}{t}$	59	-7.3	ns
	$\underset{̪}{t}$ **t**	67		
	**t ʈ**	73	-5.1	ns
	**ʈ t**	78		
	$\underset{̪}{t}$ **ʈ**	71	-6.6	ns
	**ʈ** $\underset{̪}{t}$	78		

As a visual inspection of Fig 4B suggests, the English listeners also showed patterns of perceptual asymmetry. Indeed, statistical analyses are reasonably consistent with our predictions for two of the three contrasts. The English speakers' discrimination is asymmetrical for /t $\underset{̪}{\text{t}}$/ in the /aCa/ condition, consistent with the fact that [$\underset{̪}{\text{t}}$] is a rare allophone of /t/ in English (produced in anticipatory coarticulation of a following /θ/ as in *width*), and does not contrast phonologically with [t] in the English input. However, the asymmetry found for this contrast in /##Ca/ did not reach significance, although the trend is the same. We also find support for the predicted null asymmetry for /$\underset{̪}{\text{t}}$ ʈ/. However, in neither context did the asymmetries in the perception of /t ʈ/ reach significance, although again the trend is in the predicted direction (with greater ease of discrimination when the ‘odd’ member of the pair is presented first).

#### 3.4.1. Discrimination asymmetries involving the lamino-alveopalatal

We predicted that we would not find any asymmetries involving the lamino-alveopalatal as it is acoustically and articulatorily very different from the other three coronal stops: To the non-native listeners any such contrast involving the lamino-alveopalatal and one of the three other coronals is likely to be perceived as a well-established TC contrast for which we would not predict an asymmetry. We also expected these contrasts to be highly discriminable to the native listeners, due to the pervasive acoustic and articulatory differences between the lamino-alveopalatal and the other three stops, even if the other three coronals are relatively infrequent in the lexicon and in the speech input.

To test these predictions, we again conducted a series of one-sample Wilcoxon signed rank tests of the difference in percentage correct discrimination of each contrast with regard to the two discrimination directions, for both listener groups. As predicted, the English speakers did not show any discrimination asymmetry for any of the contrasts (/ȶ $\underset{̪}{\text{t}}$/: mean difference = -1.8; /ȶ t/: mean difference = -3.0; /ȶ ʈ/: mean difference = -4.8; all non-significant), suggesting that they indeed assimilate these contrasts to English as TC contrasts.

As predicted in the case of the Wubuy listeners, we found no statistically significant difference in the percentage of correct discrimination for /ȶ ʈ/ (mean difference = 3.4, *p* = .137), but a surprising significant difference in the discrimination accuracy for both /ȶ t/ (mean diff = -6.8, *p* = .012) and /ȶ $\underset{̪}{\text{t}}$/ (mean difference = -8.03, *p* = .05), such that discrimination was easier when the highly frequent and acoustically most different (within the series) /ȶ/ was presented first. This may at first glance appear to diverge from the principle of asymmetry proposed in our own NRC hypothesis. However, it may in fact be consistent with our hypothesis, even though we had not anticipated asymmetry in these cases. Notice that an asymmetry is lacking only for the contrast in which both items are *posterior*. In the traditional approach [55], both lamino-alveopalatal /ȶ/ and retroflex /ʈ/ are classified as [—anterior] that is, with major constriction target located behind the alveolar ridge. In the remaining two contrasts, /$\underset{̪}{\text{t}}$ ȶ/ and /t ȶ/, which do show a discrimination asymmetry favouring presentation of the alveopalatal first, the other member of the pair is instead [+anterior].

These results, while surprising, are consistent with our proposal that the canonical coronal articulation is the alveolar, i.e., [+anterior]. That this anchoring effect creating a perceptual asymmetry can extend to a segment so articulatorily and acoustically distinct from the [+anterior] coronal stops as the lamino-alveopalatal is striking, particularly given the high frequency of occurrence of this stop in the input. However, it is possible that lexical frequency information is particularly relevant when the articulatory-acoustic overlap is much greater, leading listeners to recruit other kinds of information in order to make a decision about what they are hearing.

## Discussion

This paper presents two studies of native and non-native coronal consonant perception in Wubuy. To our knowledge, these studies are the first systematic examination of discrimination of a four-way coronal stop place contrast in any language to date. The results provide clear evidence that native language attunement shapes speech perception with resulting systematic differences in coronal stop discrimination between native and non-native listeners, related to both native (English vs Wubuy) coronal inventory size and frequency of occurrence of each individual coronal stop in the input for the Wubuy listeners (see 4.1 below). The results also show that, as predicted, native speech perception is not perfect when the native phonemes are acoustically and articulatorily highly similar, even when these phonemes are stops. We also find support for our suggestion that the multiple coronal stop series examined here may present a particularly difficult scenario for both native and non-native perceivers (see 4.2). Importantly, the results also address the question of what the fundamentals of consonant perception might be; indeed, they are compatible with our proposed Natural Referent Consonant Hypothesis.

### 4.1 Native language attunement and phoneme frequency shapes perception of coronal stops

#### 4.1.1 Native (Wubuy) listeners

The native Wubuy listeners' results provide new insights into the accuracy limitations of native speech perception by showing that, despite the ability of the Wubuy speakers to discriminate their native coronal stop contrasts, some contrasts (i.e., /t ʈ/, and /$\underset{̪}{\text{t}}$ ʈ/) are more difficult to discriminate than others (i.e., than those involving the lamino-alveopalatal stop) even for fluent native speakers, depending on context (word-medial or word-initial presentation). We discuss this point further below. While we did not examine the discrimination of any contrasts involving the lamino-alveopalatal in the /##Ca/ context, and therefore cannot be sure that the near-ceiling performance of contrasts involving this coronal stop in word-medial position would also be observed in initial position, the performance of the remaining three contrasts /$\underset{̪}{\text{t}}$ t/, /t ʈ/, and /$\underset{̪}{\text{t}}$ ʈ/ is similar across the two contexts (/aCa/ and /##Ca/) (see Figs 1 and 2), and suggest that we might expect ceiling performance for discrimination of contrasts involving /ȶ/ also in the /##Ca/ position. Indeed, the main effect of a decrease in discrimination accuracy from /aCa/ to /##Ca/ is due to the phoneme distribution in Wubuy: these three stops are rare in verb-stem and word-initial position, providing even native speakers with limited opportunities to tune in to the important acoustic and articulatory differences in initial position [42, 45].

This finding is reminiscent of the findings by [56] showing that English-learning infants discriminate English /ð/–/d/ rather poorly until well after their first birthday and attain adult-like performance for this contrast quite late, relative to other native contrasts. [56] explain this phenomenon with reference to the need for more input in order for the infants to successfully attune to the subtle differences between native /ð/–/d/. Notably, initial /ð/ is constrained to a single word class (determiners) in English, providing a striking parallel to systematic differences in frequency of Wubuy coronal stops discussed in [45]. Indeed, the results from our two studies with Wubuy participants, and the analyses of the frequency and positional distribution of the four coronal stops in Wubuy [45], together suggest that the characteristics of the language input play an important role in determining which contrasts are more difficult to discriminate when there is no lexical information available (i.e., in a testing situation).

The fact that some contrasts are more difficult to discriminate than others even for native listeners also addresses important yet heretofore unresolved questions in the literature on Australian languages. Research into Australian languages has typically assumed that native listeners should be able to discriminate all native contrasts in word-medial position, but that the /t ʈ/ contrast especially would be near-impossible to discriminate in initial position even for native speakers due to the lack of preceding vowel formant cues (H2d). We do not find support for this assumption: While our native speakers do appear to find the /$\underset{̪}{\text{t}}$ ʈ/ contrast difficult in initial position, this is not, statistically, the case for /t ʈ/. The Wubuy listener results thus lend perceptual support to the conclusions of [42] that place of articulation information can be carried by the *following* vowel (in addition to acoustic information in the consonant release) in Wubuy when coronal stop consonants are produced initially.

However, there may be factors at play in the case of Wubuy that make this result somewhat unusual and perhaps unexpected. There is no doubt that the majority (around 90%) of languages with an apical stop contrast neutralise it in word-initial position [10, 22]. This has been argued to be because of the difficulty of perceiving the contrast in this position [9, 22]. It is no doubt significant that Wubuy is the first language with an initial apical contrast to be tested in this way: previous studies have been conducted with languages that lacked an initial apical contrast.

In addition to the fact that Wubuy is unusual in retaining the initial apical contrast, Wubuy also differs from other Australian languages in additional ways. The overall frequency of apico-retroflex stops both within the lexicon and in discourse is quite low, compared to other Australian languages. This is no doubt partly to do with historical sound changes within Wubuy (detailed in [57]): lenis stops in the proto-language were lenited to continuants (glides and liquids), leaving few root-initial stops. Stops in the current language mostly descend from proto-language fortis stops. Fortis retroflexes are quite rare in the related languages Ngandi and Ngalakgan, and presumably were also rare in the proto-language ancestral to Wubuy.

Conversely, we might infer that the reason why apical stops are so rare root-initially in Wubuy is because of the difficulty in perceiving the contrast. Like the approximately 90% of languages with an apical contrast [10], the proto-language ancestral to Wubuy is reconstructed without an initial apical contrast [58] and Wubuy's closest congener languages (Ngandi and Enindhilyakwa) lack the contrast in this position. Contrasting apical stops were presumably re-introduced into Wubuy through borrowing. One of the neighbouring (unrelated, and undocumented) Yolngu languages, Dhay’yi, with whom the Nunggubuyu (Wubuy-speaking) people had close social ties including marriage, presumably does have an initial apical contrast like other closely related Yolngu varieties. It is possible that the contrast entered Wubuy relatively recently through this route as well as through loanwords from Makassarese in the historical period since European contact (on which see [59]), as in Yolngu languages. Neighbouring languages such as Ngalakgan and Ngandi, where the contrast is neutralised in favour of the retroflex, have a much greater proportion of retroflexes in initial position because (a) they have not lenited their initial stops, and (b) initial apicals are realised as retroflex unless there is no preceding vowel [60]. These differences mean that there are far fewer retroflexes overall in the spoken input in Wubuy than in these other languages.

In addition to the frequency factors discussed above and in greater detail in [45], the results reported here also suggest a perceptually-based distinction between the lamino-alveopalatal, on the one hand, and the remaining three coronal stops, on the other, a grouping which has hitherto not been identified in the Australianist literature. Note that this is not a natural phonological grouping: for most purposes, the set of four coronals behave phonologically like two natural classes, determined by the major articulator involved ([tip vs blade] of the tongue) (see e.g., [61, 62, 63, 10], *inter alia*). However, the two perceptually-based sets (/ȶ/ vs /$\underset{̪}{\text{t}}$ t ʈ/) cannot be distinguished in terms of that division, nor by the simple 4-way place of articulation, and we cannot use a feature such as [±anterior] to distinguish them, because the retroflex, like the alveopalatal, is regarded as [—anterior]. The only phonological feature that we might use to make this distinction is [±sharp] (following [43, 64]) as the lamino-alveopalatal has a much higher F2 locus and burst frequencies than the other three coronal stops. The distinction between [+sharp] coronal /ȶ/ and [—sharp] coronals /$\underset{̪}{\text{t}}$ t ʈ/ appears to be well-founded perceptually as well as acoustically (see also [65], for locus equation results suggesting the same distinction).

Importantly, however, there is a further respect in which these two classes might be thought of as a phonologically-motivated division. In many Australian languages (see [61, 63, 10]), apicals (/t ʈ/) are not permitted in word-initial position, and only laminals (/$\underset{̪}{\text{t}}$ ȶ/) are found. This could be thought of as a neutralisation of the three perceptually similar [—sharp] coronals /t ʈ $\underset{̪}{\text{t}}$/ in favour of the dental /$\underset{̪}{\text{t}}$/, which (in languages with this contrast) contrasts with the [+sharp] laminal /ȶ/ in this position. Similarly, in every Australian language with a within-laminal (/$\underset{̪}{\text{t}}$ ȶ/) contrast, this contrast is neutralised in pre-consonantal position (unless the following consonant is also lamino-dental; see [10], for discussion). This could in turn be thought of as a neutralisation not only of /ȶ/ with /$\underset{̪}{\text{t}}$/, but also of /$\underset{̪}{\text{t}}$/ with /t/, because /$\underset{̪}{\text{t}}$/ and /t/ do not contrast in this position. In both positions—word initially and pre-consonantally—/$\underset{̪}{\text{t}}$/ lacks one of the most salient cues to the distinction among the [**—**sharp] coronals: the much greater duration of the lamino-dental compared to the apicals [41]; see also [10] for discussion of this point). Pre-consonantally, the dental additionally lacks burst cues. Our native perception results in fact provide an explanation for these widespread patterns of neutralisation: the [**—**sharp] coronals are difficult to distinguish. The complex patterns of neutralisation and asymmetric frequency that we find in Australian languages argue for a more nuanced view of the phonological behaviour of the coronals, and for a fuller consideration of the role of 'canonical' consonants as natural 'anchors' for speech perception.

In sum, the Wubuy results allow us to reject the first hypothesis (H1), that all Wubuy coronal stop contrasts will be discriminated equally well. The results are consistent with the second hypothesis (H2) that native phoneme discrimination is subject to factors such as acoustic and/or articulatory similarity/overlap, consonant frequency, and consonant distribution in the input. Indeed, despite the fact that previous work on the acoustics of Wubuy stops [42] found less acoustic evidence to support the /t $\underset{̪}{\text{t}}$/ contrast than the other contrasts, native listeners clearly found this contrast relatively easy to discriminate: it did not differ significantly from contrasts involving the lamino-alveopalatal, nor did it suffer in terms of discriminability by presentation in word-initial position, perhaps indicating that input frequency trumps acoustics.

#### 4.1.2 Non-native (English) listeners

The English listeners were successful in their discrimination of contrasts involving the lamino-alveopalatal /ȶ/, but they found the /$\underset{̪}{\text{t}}$ t/, /t ʈ/, and /$\underset{̪}{\text{t}}$ ʈ/ contrasts more challenging, to varying degrees. Indeed, in the /aCa/ context, the English listeners had more difficulty than the Wubuy listeners—they found the discrimination of /t $\underset{̪}{\text{t}}$/ and /ʈ t/ difficult, yet did well in the discrimination of the acoustically more well-differentiated /$\underset{̪}{\text{t}}$ ʈ/. Their discrimination performance in the /##Ca/ context, on the other hand—and unlike the Wubuy listeners—improved over their /aCa/ performance. Discrimination of /$\underset{̪}{\text{t}}$ t/ however was also difficult in this context compared to discrimination of /t ʈ/ and /$\underset{̪}{\text{t}}$ ʈ/.

The findings support our PAM/PAM-L2 predictions that English listeners should perceive contrasts involving the lamino-alveopalatal as TC assimilations, and the remaining three contrasts (/$\underset{̪}{\text{t}}$ t/, /t ʈ/, and /$\underset{̪}{\text{t}}$ ʈ/) as CG contrasts in which /t/ is perceived as a ‘good’ English /t/ and /$\underset{̪}{\text{t}}$/ and /ʈ/ are perceived as ‘odd’ or ‘deviant’ English /t/s. This is consistent with the difficulty in discriminating French (dental and prevoiced) /d/ and English (alveolar and short-lag voiced) /d/ experienced by native monolingual speakers of French and English [51]. The fact that there is no significant difference in relative discriminability of /$\underset{̪}{\text{t}}$ t/ and /t ʈ/ in the /aCa/ context may indicate that, at least in medial position, /$\underset{̪}{\text{t}}$/ and /ʈ/ may be perceived as equally ‘odd’ English /t/s by the English listeners. We further suggest that the improved performance from the /aCa/ context to the /##Ca/ context is most probably a reflection of these non-native listeners’ increased familiarity with the previously-unexperienced Wubuy phones over the course of the experiment, in which they completed the /aCa/ context before they went on to the /##Ca/ context. Finally, we suggest that the difference in discrimination accuracy for /$\underset{̪}{\text{t}}$ t/ and /t ʈ/ in /##Ca/ may indicate that the English listeners may be re-tuning their perception to those two contrasts at different rates. Indeed, English listeners may be able to tune into /t ʈ/ due to their relative familiarity with rhotic /r/ in American English TV and movies, in which the /t/ tongue tip contact in words such as *parting* and *carton* may be somewhat more posterior (~ more ‘retroflexed’) than in the corresponding words *potting* and *cotton*. Even though American English does not allow initial /rC/ clusters, other research [66] has shown that the location of the acoustic and articulatory correlates of English /r/ can be difficult for listeners to pinpoint. Indeed, /r/ is often perceived to spread throughout neighbouring syllables, even potentially to initial syllables, giving speakers the phenomenological sense of hearing /rC/, and perhaps allowing them to successfully discriminate /t ʈ/ in initial position, despite the lack of actual experience with retroflexes in this position.

#### 4.1.3 Native and non-native comparison

The relative performance levels of the Wubuy and English listeners indicate, unsurprisingly, that the native listeners found the task easier than the non-native listeners in many respects. In the /aCa/ context, the native listeners outperformed the non-native listeners on /$\underset{̪}{\text{t}}$ ʈ/, and in both contexts they outperformed the non-native listeners on /$\underset{̪}{\text{t}}$ t/ (though the difference in the /##Ca/ context only approached significance). This is, of course, hardly unexpected, given the respective phonological systems of the native and non-native participants. Interestingly, however, the non-native listeners were on par with the native listeners in their discrimination of the /t ʈ/ contrast in the /aCa/ context, and actually outperformed them in the /##Ca/ context. We believe the explanation for this is two-fold. Firstly, the native listeners’ pattern is consistent with the low frequency of the retroflex across all word positions in Wubuy, and in particular the near-absence of the retroflex in word- and verb stem-initial position, which is associated with a relatively poor discrimination performance when there is no lexical information to assist the listener, as was the case in our XAB task. Secondly, we attribute the improved non-native listener results in the /##Ca/ context to improved perceptual tuning and performance on task; these listeners are already familiar with retroflex articulations through media exposure to the 'rhotic' American English accent and may have simply improved their accuracy through exposure. By contrast, the native listeners presumably cannot improve their performance, in such a short-duration artificial task, on acoustic stimuli that already represent native phonemic categories for them.

The comparison between the Wubuy and the English speaker groups, however, deserves a number of comments. Firstly, we would like to highlight the fact that statistical analyses such as ours are based on the assumption that any differences between the groups on the various measures are due entirely to the single variable compared, here the group factor of native versus non-native listeners. We do not believe that the results from the present study are perfectly balanced in this respect, though, as the two participant groups differ in (at least) two crucial aspects, outlined below, that are likely to affect their general performance.

Firstly, these two groups differ greatly in their computer and task literacy. The non-native listeners were all highly educated and well-skilled in computer use, as well as familiar with experimental paradigms in general and, for most of them, linguistic research in particular. Several participants had previously partaken in other (unrelated) language research conducted by the first, fourth and fifth author. The Wubuy participants, on the other hand, had only very basic computer skills and experience, including numeracy (recall that they had to respond by pressing either the ‘2’ or ‘3’ key on a keyboard). Secondly, the two participant groups also differ greatly in their overall literacy. While the English participants were highly literate, most of the Wubuy participants, as is typical of remote Aboriginal populations (e.g., [67]), had limited literacy in both their native language and in English. While all were able to sign consent forms, as required, most preferred to have the forms read out to them and then translated into oral Wubuy, rather than reading them. This was also the case with the language background questionnaire. It is possible that this lower literacy level in the Wubuy participant group results in a lower degree of phonemic awareness, though we did not test their phonemic awareness to examine this directly. Finally, we would like to also highlight the difference in mean age of the two groups. While we did not test the participants’ hearing, it is possible that age-related hearing-loss may have played a role for the Wubuy speakers, especially given the high prevalence of otitis media in remote Australian communities.

While we believe that the patterns of ease/difficulty of discrimination observed for the native Wubuy listeners reflects the relative ease/difficulty of discriminating the contrasts, and the results are in line with the predictions set out in the introduction, we also acknowledge that their overall discrimination scores are likely to reflect a greater difficulty with the task (due to relative inexperience with computers and experimental testing, and perhaps greater inherent task difficulty due to lower phonemic awareness, literacy and numeracy) for the following observable reasons: (1) We were unable to explain the experimental task to approximately 33% of the recruited Wubuy participants, despite a task-literate native Wubuy-speaking assistant helping the first and second authors with explaining the task, and generous practice time. We did not have this difficulty with the English participants: all participants understood the task easily and had no difficulty remembering or following the instructions given. (2) We observed a lower range of discrimination scores for the Wubuy listeners than the English listeners for the contrasts involving /ȶ/, despite the lack of any indication from the existing literature on coronal stops in Australian languages that contrasts involving the lamino-alveopalatal are difficult for native *or* non-native listeners to discriminate from any of the other coronal stops. (3) We observed greater variation among the native Wubuy speakers in terms of discrimination accuracy, than within the English-speaking group. Furthermore (4), we note that those Wubuy participants who did particularly well on the perception tasks were also those who were the most literate and the most computer-skilled.

Unfortunately, the large within-group differences in literacy and computer skills observed in our Wubuy participant group are virtually unavoidable. The limited number and advanced age of speakers of this language (~60, most past the age of 55) alone imposes great restrictions on participant selection. So does the geographical spread of the population: while many Wubuy speakers live in Numbulwar (on the Gulf of Carpentaria), some live in Darwin, nearly 800 km away, for medical or other reasons, or Groote Eylandt, a plane flight away from Numbulwar across the Gulf. Social and cultural norms and expectations also restrict selection as some speakers may be unwilling to participate in language work for a number of reasons (e.g., they may be reticent to work with unfamiliar non-Indigenous researchers; may not feel that they have the right level of competence in their native language; or may be expected to be elsewhere).

### 4.2 The Natural Referent Consonant Hypothesis

Our analysis of the asymmetries in the discrimination patterns of the native and non-native speakers is consistent with the predictions of the NRC hypothesis that the three major articulators (lips, tongue front, tongue back) may provide perceptual and articulatory ‘anchor points’, corresponding to the three major oral places of articulation: labial, coronal, and dorsal. Our findings are also consistent with the NRC hypothesis' fundamental assumptions that 1) the most canonical articulation will be the perceptual anchor in consonant discrimination; 2) the canonical articulation for the coronal place is apical orientation of the tongue tip, with contact made in the alveolar region; and 3) consonant contrasts may continue to display an innate bias towards the Natural Referent Consonant if the canonical articulation is also the more frequent member of the contrast.

We hope our framework and the studies presented here invite more research to test the NRC and its predictions. Indeed, the perceptual asymmetries observed here must be accounted for, and the NRC provides the only current testable framework. We recognise, however, that while our results are consistent with the NRC hypothesis, we cannot conclusively determine whether the discrimination behaviour of the two listener groups in our studies is due to language experience alone, or if the Natural Referent Consonant (or, perhaps more precisely, Natural Referent Place of Articulation) principles play a role in the discrimination patterns observed. In the case of the English listeners, the NRC hypothesis predicts a pattern of behaviour identical to that predicted by PAM on the basis of language specific learning of coronal consonants: /t/ would be expected to be canonical in this group, resulting in the observed asymmetries, both on the basis of language specific learning of English, and on the basis of the alveolar place of articulation being the canonical or default setting. This pattern is exactly what we observed: canonical /t/ behaves as a perceptual anchor for English speakers in the discrimination of /t/ versus /$\underset{̪}{\text{t}}$/ and /ʈ/.

In the case of the Wubuy listeners, we observed that the discrimination of frequently-occurring native phonological categories is symmetrical, even for coronal stops with a POA other than alveolar, e.g. the lamino-dental stop. This finding is compatible with the findings reported in [12, 13] that native language acquisition can override the initial Natural Referent Vowel effects of better discrimination when a less peripheral vowel is presented prior to presenting a more peripheral vowel, observed in infant speech perception and in cross-language/L2 perception. The fact that /ȶ/ does not act like an anchor, despite its frequency of occurrence, is likely due to the fact that it is not /t/-like, as opposed to /$\underset{̪}{\text{t}}$/ and /ʈ/, and easily discriminated (despite the asymmetry) by native and non-native listeners alike (see also [9]). Our results, however, also showed that perceptual asymmetries do exist even for native speakers in the special situation when one of two phonemes in a contrast is infrequent in the input. This has not been explicitly considered for native vowel perception, nor been examined experimentally, but may well apply to vowels also. In any case, our findings support the notion of a NRC bias that will only disappear if native language experience provides sufficient input for the relevant phonological contrasts to be established and reinforced. And this is arguably not the case for Wubuy /ʈ/ which is exceedingly rare in natural Wubuy input. To conclusively tease apart language specific attunement factors and a potential NRC in the context of the present study, however, we would have to present our two tasks to speakers of a language with a dental and a retroflex stop, but no alveolar stop. Here, we would expect native language attunement to override any NRC effects so that /ʈ/ followed by /t/ and /$\underset{̪}{\text{t}}$/ followed by /t/ would always be discriminated at the same level of success as /t/ followed by /ʈ/ and /t/ followed by /$\underset{̪}{\text{t}}$/. Hindi, as well as other Indo-Iranian and Dravidian languages of South Asia, have these characteristics and we look forward to future studies of these L1 listeners’ perception of Wubuy coronal stops.
